# Prophylaxis and Treatment against *Klebsiella pneumoniae*: Current Insights on This Emerging Anti-Microbial Resistant Global Threat

**DOI:** 10.3390/ijms22084042

**Published:** 2021-04-14

**Authors:** Vanessa Arato, Maria Michelina Raso, Gianmarco Gasperini, Francesco Berlanda Scorza, Francesca Micoli

**Affiliations:** GSK Vaccines Institute for Global Health (GVGH) S.r.l., via Fiorentina 1, 53100 Siena, Italy; vanessa.x.arato@gsk.com (V.A.); maria-michelina.m.raso@gsk.com (M.M.R.); gianmarco.x.gasperini@gsk.com (G.G.); francesco.x.berlandascorza@gsk.com (F.B.S.)

**Keywords:** *Klebsiella pneumoniae*, anti-microbial resistance, vaccines, monoclonal antibodies

## Abstract

*Klebsiella pneumoniae* (Kp) is an opportunistic pathogen and the leading cause of healthcare-associated infections, mostly affecting subjects with compromised immune systems or suffering from concurrent bacterial infections. However, the dramatic increase in hypervirulent strains and the emergence of new multidrug-resistant clones resulted in Kp occurrence among previously healthy people and in increased morbidity and mortality, including neonatal sepsis and death across low- and middle-income countries. As a consequence, carbapenem-resistant and extended spectrum β-lactamase-producing Kp have been prioritized as a critical anti-microbial resistance threat by the World Health Organization and this has renewed the interest of the scientific community in developing a vaccine as well as treatments alternative to the now ineffective antibiotics. Capsule polysaccharide is the most important virulence factor of Kp and plays major roles in the pathogenesis but its high variability (more than 100 different types have been reported) makes the identification of a universal treatment or prevention strategy very challenging. However, less variable virulence factors such as the O-Antigen, outer membrane proteins as fimbriae and siderophores might also be key players in the fight against Kp infections. Here, we review elements of the current status of the epidemiology and the molecular pathogenesis of Kp and explore specific bacterial antigens as potential targets for both prophylactic and therapeutic solutions.

## 1. Introduction

Multidrug-resistant (MDR) bacteria are defined as bacteria resistant to more than three antibiotic classes [1]. In February 2017, the World Health Organization (WHO) published a list of MDR pathogens to focus the research and development pipeline of new antibiotic treatments. Within this broad list, *Enterococcus faecium*, *Staphylococcus aureus*, *Klebsiella pneumoniae*, *Acinetobacter baumannii*, *Pseudomonas aeruginosa*, *Enterobacter* species and *Escherichia coli* (ESKAPEE) pathogens were given the highest priority status [2]. Indeed, ESKAPEE pathogens have developed resistance mechanisms against most antibiotic treatments including those that are the last line of defense such as carbapenems and polymixins [3].

*Klebsiella pneumoniae* (Kp) was first identified as a causative agent of pneumonia in 1882 and has constantly gained significant public health attention. During the 1960s and 1970s, Kp became one of the most important causes of opportunistic healthcare-associated infections. In addition to pneumonia, Kp can also cause infections in the urinary tract and lower biliary tract, infections in surgical wound sites as well as bloodstream-associated infections [4]. The most vulnerable patients are neonates and the elderly, especially those that are immunocompromised. 

Kp is naturally equipped with enzymes able to hydrolyze β-lactam antibiotics such as ampicillin and amoxicillin. However, the genome of Kp continued to accumulate antimicrobial resistance (AMR) genes through acquisition of plasmids and transferable genetic elements [5]. To date, over 400 AMR genes have been identified in different Kp genomes [6]. The introduction in the early 1980s of the third generation cephalosporins to treat ampicillin-resistant *Enterobacteriaceae* resulted in the emergence of extended-spectrum β-lactamase Kp (ESBL-Kp), which are currently prevalent worldwide and most frequently linked to high mortality rates [7,8]. Carbapenems were then the drug of choice for the treatment of ESBL-Kp in the 1990s, but this has resulted in the emergence of carbapenem-resistant Kp (CR-Kp), which have been alarmingly increasing in incidence since then [8,9]. It is noteworthy that a case–control study, conducted in two different hospitals in Greece, showed no statistical difference in hospital mortality between patients with CR-Kp (30.1%) and patients with carbapenem-susceptible Kp (CS-Kp) (33.9%). However, this may be due to the significant use of polymyxins for the treatment of severe multidrug-resistant infections in these hospitals, suggesting that the mortality of the CR-Kp could be possibly higher, if polymyxins were not used [10]. 

Different AMR mechanisms have been described in CR-Kp, including acquired carbapenemases (KPC), New Delhi metallo-β-lactamase (NDM) and carbapenemases of the oxacillinase-48 (OXA-48) type [11]. KPC-producing Kp has been most commonly occurring in the United States, after its first isolation in North Carolina [12,13]. NDM-producing Kp has been most commonly associated with the Indian subcontinent, since after its first detection in New Delhi [14], but also with specific countries in Europe, including Ireland [15], the Netherlands [16] and Italy [17]. The epicenter of OXA-48-like-producing Kp is in Turkey and surrounding countries [18], however health care-associated outbreaks have also been recently described in various European countries, including Greece [19], Ireland [20], France [21] and Germany [22], as well as in China [23] and Taiwan [24].

Beside the role of opportunistic pathogen in hospital settings, Kp can cause severe community-acquired infections in otherwise healthy individuals, such as pyrogenic liver abscess and meningitis often accompanied by bacteremia and metastatic spread [25]. Kp strains able to cause such infections are recognized as hypervirulent Kp (Hv-Kp) and were first detected in the 1980s in Taiwan [26], Hong Kong [27] and South Korea [28]. Hypervirulence-associated genes are often encoded on a large virulence plasmid but can also be encoded in the chromosome as part of integrative conjugative elements [29]. Such genes include *rmpA*/*rmpA2* (associated to the hypermucoviscous phenotype), *iroBCDN*, *iutA*, *iuacABCD* and *ybt* genes (encoding multiple siderophores and their receptors) and the *clb* locus (encoding the colibactin genotoxin) [30,31,32].

Interestingly, MDR-Kp and Hv-Kp evolved from two different directions resulting in largely non-overlapping phenotypes [33]. Indeed, MDR-Kp clones maintained low virulence while Hv-Kp remained susceptible to carbapenems. The reasons for the apparent separation of phenotypes are still unclear, however growing reports of bi-directional convergence represent an alarming threat to public health [34,35,36]. 

The rising rates of AMR have a substantial economic impact globally, even though it remains difficult to be accurately calculated. This is related to extended duration of hospitalization and higher costs of new drugs to treat the infections. Low- and middle-income countries (LMIC) are already affected by AMR and are considered to be at higher risk of additional morbidity and mortality in the near future [37,38,39]. In particular, MDR bacterial infections are a leading cause of neonatal sepsis which accounts for over one-third of the global burden of child mortality [40]. Gram-negative rods are major pathogens of neonatal sepsis in LMIC and Kp is responsible for 16–28% of blood-culture-confirmed sepsis in different regions of the world. Assuming conservatively that Kp is responsible for 20% of the estimated 1.6 million neonatal sepsis-related deaths in the developing world, Kp-related neonatal deaths could amount to 320,000 deaths per year [40]. Child mortality remains disproportionately high in sub-Saharan Africa and South Asia [41]. A systematic review and meta-analysis of available data from the African continent, with a focus on the past decade (2008–2018), identified *Klebsiella* spp (mostly Kp) as accountable for 21% of all reported cases of neonatal bacteremia or sepsis [42]. The Child Health and Mortality Prevention Surveillance (CHAMPS) Network, established to collect data on under-5 mortality and stillbirths in sub-Saharan Africa and South Asia, also identified Kp as the most common contributory pathogen of neonatal deaths with at least one infectious condition in the causal chain, together with *Acinetobacter baumannii* and *E. coli* [43]. While more studies, including the Burden of Antibiotic Resistance in Neonates from Developing Societies (BARNARDS) study and the Global Neonatal Sepsis Observational Study (NeoOBS), are currently collecting more field data, the available estimates clearly highlight the need to acknowledge Kp as one of the most important neonatal pathogens in LMIC. 

## 2. *Klebsiella pneumoniae* Diversity

The genus *Klebsiella* was named after the German microbiologist Edwin Klebs and is part of the *Enterobacteriaceae* family. However, it was termed *Friedlander bacillus* for many years in honor of the German pathologist Carl Friedländer, who first described this bacterium as the causative agent of pneumonia in immunocompromised patients. Members of this genus are Gram-negative rod-shaped bacteria that are found everywhere in nature, including water, soil, plant, insects and animals [44]. In humans, members of the genus *Klebsiella* are part of the normal flora in the nose, mouth and intestines. The species *Klebsiella pneumoniae* was first distinguished from other species of the genus *Klebsiella* on the basis of *gyrA* and *parC* genes and it was divided into three subspecies named *K. pneumoniae subsp. pneumoniae*, *K. pneumoniae subsp. ozaenae* and *K. pneumoniae subsp. rhinoscleromatis* [45]. However, closely related species of the genus *Klebsiella* might share 95–96% average nucleotide identity with Kp and only the implementation of whole-genome sequencing (WGS) facilitated the correct identification of clinical isolates [46].

Phenotypic methods developed for typing Kp isolates include phage typing, bacteriocin typing and serotyping. However, multilocus sequence typing (MLST) was first used by Diancourt et al., to accurately describe the genetic relationships among Kp isolates [47]. The MLST scheme developed was based on the sequences of seven housekeeping genes and allowed one to differentiate forty distinct allelic profiles, also known as sequence types (ST). A different MLST scheme, based on the sequences of 694 highly conserved core genes, was later developed by Bialek-Davene et al. and enabled the precise definition of globally distributed Kp clonal groups (CG) [48]. Most recently, WGS allowed one to show the existence of >150 deeply branching Kp lineages [31]. Whether defined as lineages, CG or ST, the resulting groups are typically referred to as clones. Kp infections are caused by different clones worldwide, but only a subset of these clones is responsible for the global disease burden. MDR-Kp clones are included in the largely described CG258, together with CG15, CG20, CG29, CG37, CG147, CG101 and CG307 [49,50]. In contrast, Hv-Kp clones are included in CG23, together with CG25, CG66 and CG380 [51,52]. While MDR-Kp CGs are each widely geographically distributed, Hv-Kp CGs are highly prevalent in Asia and the Pacific rim [53]. 

Historically, Kp has been classified into serotypes and tracked using typing antisera. Kp produces two types of polysaccharide on the cell surface: capsular polysaccharides (K-antigens) and O-antigens as part of the lipopolysaccharide (LPS) [54]. Both contribute to pathogenicity and form the basis for the identification of isolates, or serotyping. The most recently developed serotyping method relies on the genetic sequence analysis of the *cps* locus (K-antigen) and the *rfb* locus (O-antigen) [55]. To date, 141 distinct K-antigens have been identified, however, only 77 of these have been distinguished by traditional serological typing [56]. A substantial K-antigen diversity is observed in MDR-Kp clones, while K1 and K2 antigens are strongly associated to Hv-Kp clones [54]. In contrast, Kp has a surprisingly low number of reported O-antigens and only 11 distinct serotypes have been described. O1, O2, O3 and O5 are most commonly found in Kp clinical isolates, despite the fact that the prevalence of each serotype can vary based on the geographical regions and the MDR or Hv phenotypes [54,57,58].

## 3. *Klebsiella pneumoniae* Virulence Factors and Key Target Antigens

**Figure 1:**
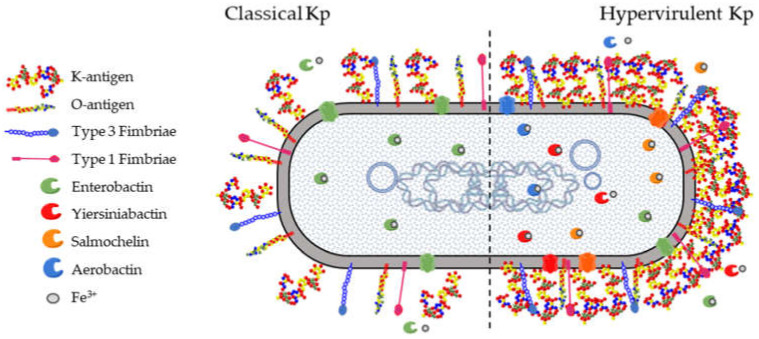
*K. pneumoniae* virulence factors. There are four well-characterized virulence factors for pathogenic *Klebsiella pneumoniae* (Kp). (1) The capsule is an extracellular polysaccharide matrix that envelops the bacteria and is overproduced in hypervirulent Kp (Hv-Kp) strains. (2) Lipopolysaccharide (LPS) is an integral part of the outer leaflet of the outer membrane and is produced by both classical and Hv-Kp strains. (3) Type 1 and type 3 fimbriae are membrane-bound adhesive structures. (4) Iron-scavenging siderophores are secreted small molecules recognized by specific membrane receptors mediating their uptake. Enterobactin is produced by virtually all Kp strains while other siderophores are typically secreted by Hv-Kp strains.

All Kp isolates carry a set of core genes responsible for their pathogenicity and required to establish opportunistic infections in humans and other hosts (Figure 1). These include the K-antigen locus (*cps* locus) and the O-antigen locus (*rfb* locus), collectively involved in immune evasion mechanisms. The core *fim* and *mrk* loci are responsible for the biosynthesis of type 1 and type 3 fimbriae which mediate processes in the earlier stages of infection, such as adhesion and colonization of host epithelia as well as biofilm formation on abiotic surfaces such as catheters. Finally, the *ent* locus encoding the siderophore enterobactin, as well as alternative acquired siderophores, is required for optimal growth in different niches.

### 3.1. K-Antigens

The surface of *Klebsiella* species is shielded by a thick layer of capsular polysaccharide, historically known as K-antigen, that protects the bacteria from the environment as also observed in *E. coli* [59]. K-antigens play a crucial role in protecting Kp from innate immune response mechanisms, evading complement deposition and opsonization, reducing recognition and adhesion to epithelial cells and phagocytes, and abrogating lysis by antimicrobial peptides and the complement cascade [60]. 

Traditionally, 77 K-antigens have been identified among *Klebsiella* spp. based on the diversity in their sugar composition, type of glycosidic linkages, and the nature of enantiomeric and epimeric forms [61,62]. Recently, additional K-types have been reported based on the arrangement of the *cps* locus or K-locus (KL), known as the KL series [56]. 

**Figure 2:**
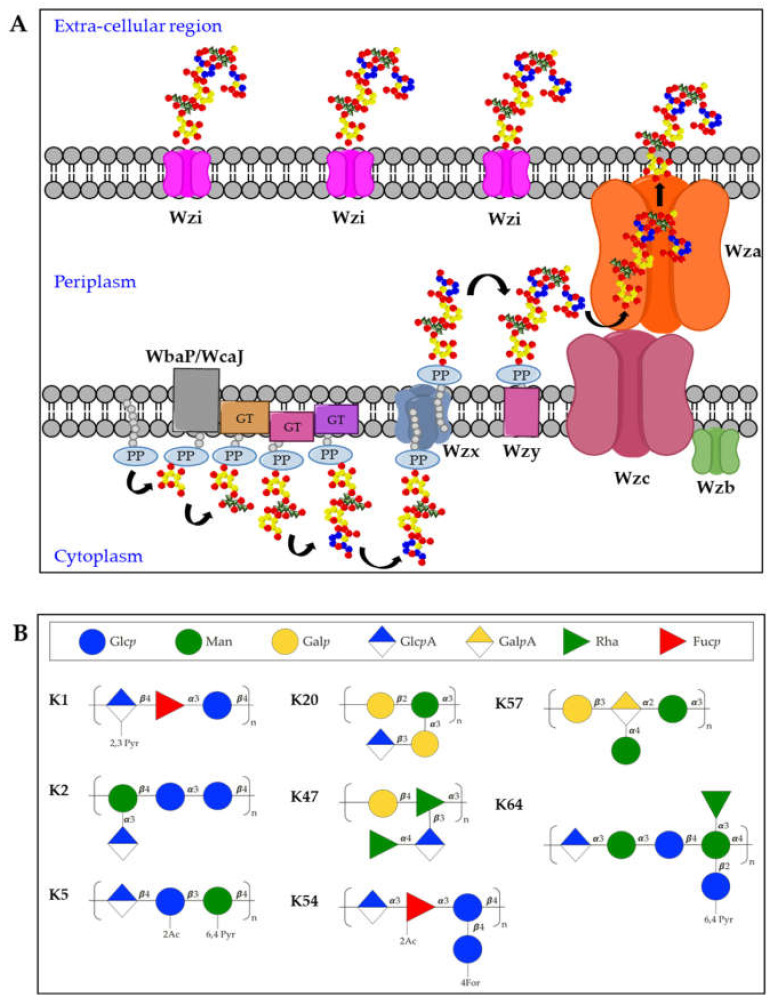
(**A**) Model for biosynthesis and assembly of group 1 capsules. Undecaprenol-pyrophosphate (Und-PP)-linked repeating units are assembled on the cytoplasmic leaflet of the inner membrane. Wzx then flips the newly synthesized und-PP-linked repeats across the inner membrane. In the periplasmic leaflet of the inner membrane, Wzy polymerizes the repeating units. Continued polymerization requires transphosphorylation of the Wzc oligomer and dephosphorylation by the Wzb phosphatase. Finally, the polysaccharide is translocated by Wza in the extracellular milieu where it associates with the surface protein Wzi. PP = Undecaprenyl diphosphate, GT = glycosyltransferase. (**B**) Structures of K-antigens most commonly associated to Hv-Kp strains.

The K-antigen biosynthesis occurs via the Wzx/Wzy-dependent pathway, similarly to group 1 capsules in *E. coli* [63,64]. The *cps* locus comprises two regions: the 5′ part of the locus contains four conserved genes (*wzi*, *wza*, *wzb*, and *wzc*), present in all group 1 capsule loci. The 3′ region of the locus is serotype-specific and encodes the enzymes for producing the repeating units and two integral inner membrane proteins (Wzy and Wzx) [65]. In particular, capsules biosynthesis takes place in the cytoplasmic leaflet of the inner membrane where the repeating units are assembled on the lipid carrier undecaprenol-pyrophosphate (Und-PP), with the contribution of sugar-specific glycosyltransferases. In particular, the specific initialization glycosyltransferase can either be WbaP (generating galactose-Und-PP) or WcaJ (generating glucose-Und-PP) [66]. Next, the Wzx flippase transfers the complete repeating units to the periplasmic leaflet of the inner membrane where the Wzy polymerase generates high molecular weight polysaccharides. Finally, Wza (an outer-membrane translocon), Wzc (a tyrosin autokinase) and Wzb (a phosphatase) synergistically transport the complete capsule onto the bacterial surface, where it remains associated to the outer-membrane protein Wzi (a lecto-aqua-porin) [62] (Figure 2A). 

Among the different K-antigens, K1, K2, K5, K16, K23, K27, K28, K54, K62 and K64 are some of the most commonly isolated serotypes globally [54]. Interestingly, K1 and K2 serotypes are often found in Hv-Kp strains, indicating that this clonal lineage has a specific genetic background conferring hypervirulence [67,68] (Figure 2B). Moreover, Hv-Kp strains are known to produce a hypercapsule, resulting in a hypermucoviscous phenotype which further contribute to increased resistance to complement-mediated or phagocyte-mediated killing [69]. The *rmpA* gene was first identified as a regulator of the mucoid phenotype in 1989 [70], located either on the chromosome or on a large virulence plasmid. The correlation between *rmpA* (or the closely related *rmpA2* gene) and hypermucoid phenotype is very high, and the presence of *rmpA* is among a set of genes proposed as biomarkers to identify potential Hv-Kp strains [29,71]. Other capsule types which are frequently found among Hv-Kp strains are K5, K20, K47, K54, K57, and K64 [29,72,73] (Figure 2B).

### 3.2. O-Antigens

The O-antigen moiety of the LPS has a limited range of structures, resulting from different sugar composition, glycosidic linkages, epimeric or enantiomeric forms of the sugars [54,74]. The nomenclature of O-serotypes has been revised multiple times over the years [75,76], however, the most recent classification includes 11 O-serotypes: O1, O2a, O2ac, O2afg, O2aeh (previously known as O9), O3 (divided in sub-serotypes O3, O3a and O3b), O4, O5, O7, O8 and O12 [56]. Moreover, additional O-types have been reported based on the arrangement of the *rfb* locus or O-locus (OL), known as the OL series. 

**Figure 3:**
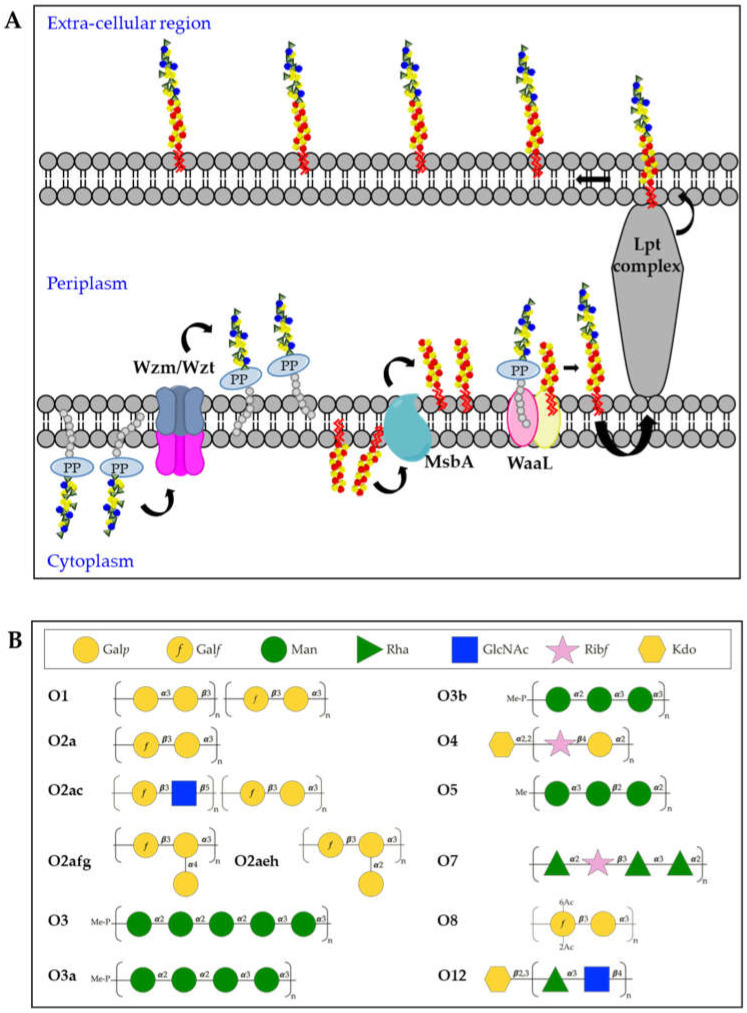
(**A**) Model of O-antigen biosynthesis via the adenosine triphosphate-binding cassette (ABC) transporter pathway. Und-PP-linked O-polyaccharides are processively assembled on the cytoplasmic leaflet of the inner membrane, through the sequential activity of different glycosyltransferases. The assembled O-antigen component is then transported via the Wzm/Wzt ABC transporter across the inner membrane. In the periplasmic leaflet of the inner membrane, the O-antigen is ligated to the lipid A-core oligosaccharide by WaaL and finally transported on the bacterial surface by the Lpt complex. (**B**) Structures of Kp O-antigens.

Kp O-antigens are biosynthesized in the cytoplasm and transported to the bacterial surface through an adenosine triphosphate (ATP)-binding cassette (ABC) transporter dependent-pathway [77] (Figure 3A). In detail, the O-antigen is synthesized in the cytoplasmic leaflet of the inner membrane by serotype-specific glycosyltransferases and transferred to the periplasmic leaflet by the Wzm/Wzt complex. In parallel, the biosynthesis of the lipid A-core oligosaccharide also takes place in the cytoplasmic leaflet of the inner membrane and the sugars are then flipped to the periplasmic leaflet through the MsbA transporter. Finally, the polymerized O-antigen and lipid A-core oligosaccharide are linked by the WaaL ligase and the LptA-G complex transports the complete LPS to the bacterial surface [62]. In the whole process, three different gene clusters are involved: *lpx*, *waa* and *rfb* for the biosynthesis of lipid A, core-oligosaccharide and O-antigens, respectively [78,79,80].

The O1, O2 and O8 serotypes are galactose-based polysaccharides and they share a common backbone named O2a (also known as Galactan-I) characterized by the [→3)-α-D-Gal*p*-(1 → 3)-β-D-Gal*f*(1→] repeating unit (Figure 3B). The biosynthesis of the O2a antigen is attributed to the *rfb* locus involving *wzm*, *wzt*, *wbbM*, *glf*, *wbbN*, and *wbbO* genes [81]. The O2 serotype includes different variants, represented by modified versions of the O2a backbone. The O2afg and O2aeh (O9) serotypes modify the O2a repeating unit by side-chain addition of (α-1 → 4)- or (α-1 → 2)-Gal*p* residues catalyzed by a set of three enzymes encoded by genes *gmlABC* and *gmlABD*, respectively [82]. The O8 serotype modifies the O2a repeating unit by non-stoichiometric O-acetylation [83] (Figure 3B). The O1 serotype results from the covalent attachment of the O1 antigen (also known as Galactan-II) to the non-reducing terminus of O2a (as well as O2afg and O2aeh) and is characterized by the immunodominant [→3)-α-D-Gal*p*-(1→3)-β-D-Gal*p*-(1→] repeating unit, which has been associated to the *wbbY-wbbZ* genes [84] (Figure 3B). The O2c antigen can also be covalently attached to the non-reducing terminus of O2a (as well as O2afg and O2aeh), resulting in serotype O2ac: this is characterized by the immunodominant [→3)-β-D-Glc*p*NAc-(1 → 5)-β-D-Gal*f*-(1→] repeating unit, which has been associated to the *wbmVWX* genes [84] (Figure 3B). 

The O3 and O5 serotypes are mannose-based saccharides and their structures are identical to the *E. coli* O9 and O8 O-antigens, respectively [85,86] (Figure 3B). Biosynthetic enzymes of O3 and O5 are extremely similar but differ in the sequence of their mannosyltransferase (WbdA) and methyltransferase (WbdD). WbdD, in complex with WbdA, regulates the mannose chain length by capping the growing chain with a phosphate and methyl group in O3 and a methyl group only in O5 [87,88]. Therefore, the O3 and O5 repeating units differ from each other in the number of mannoses, anomeric configurations and/or their intra-mannose linkages [89]. In particular, the trimeric O5 repeating unit is composed of α-mannose (α-Man) and β-mannose (β-Man) with 1 → 2 and 1 → 3 linkage, whereas O3 uses pentameric 1 → 2- and 1 → 3-linked α-Man repeating units [89]. In addition, two Kp O3 serotype variants, O3a and O3b, have been identified with only four α-Man residues (O3a) or three α-Man residues (O3b), instead of five α-Man residues per repeating unit [90] (Figure 3B).

The O4, O7 and O12 serotypes are very different to the other O-antigens for their repeating units sugar composition (Figure 3B). The Kp O4 repeating unit is characterized by the disaccharide [→4)-α-D-Gal*p*-(1 → 2)-β-D-Rib*f*-(1→], the O7 by a tetrasaccharide [→2)-α-L-Rha*p*-(1 → 2)-β-D-Rib*f*-(1 → 3)-α-L-Rha*p*-(1 → 3)-α-L-Rha*p*-(1→] while the O12 by the disaccharide [→4)α-L-Rha*p*-(1 → 3)-β-D-Glc*p*NAc-(1→] [89]. In addition, it was found that the O4 and O12 O-antigen chains terminate with Kdo residues. 

### 3.3. Fimbriae

The majority of Gram-negative enterobacteria differentially express surface-associated fimbriae, organelles appointed to facilitate attachment and adherence to eukaryotic cells, but also involved in other functions, such as interaction with macrophages, biofilm formation, intestinal persistence, and bacterial aggregation [91,92,93]. Fimbriae are typically extracellular appendages with 0.5–10 μm length and 2–8 nm width. Most clinical Kp isolates express two types of fimbrial adhesins, type 1 fimbriae and type 3 fimbriae, that are assembled by the chaperone/usher-assembly pathway [94,95,96]. Besides type 1 and type 3 fimbriae, a third type named KPC fimbria was identified and the heterologous expression in *E. coli* demonstrated an active role of this protein in biofilm formation [97]. Since the late 1990s, another Kp fimbrial antigen named KPF-28 has been reported to be expressed by several Kp circulating strains and its role in colonization has been demonstrated by using KPF-28 antisera to inhibit bacterial adhesion to intestinal cells. However, besides a correlation of the expression of this adhesin with an antibiotic-resistance phenotype, no further information has been collected about KPF-28 over the last two decades [98,99]. 

Type 1 fimbriae are 7 nm wide and approximately 1 µm long surface polymers found on the majority of *Enterobacteriaceae* and encoded by the genes in the *fimAICDFGHK* operon. This organelle is a helical cylinder that belongs to the chaperon-usher pili family, made by a polymer of the major building element FimA. FimH, together with the minor subunits FimF and FimG, forms a flexible tip fibrillum that is connected to the distal end of the pilus rod and that is responsible of the adhesion to the host [100,101,102,103]. FimH recognizes mannosylated glycoproteins, including those present on the host urinary epithelium as demonstrated by using D-mannose or oligosaccharides containing terminal mannose residues in FimH-mediated adhesion inhibition experiments [104,105].

Type 1 fimbriae were described to be phase-variable with a different role in lungs and urinary or intestinal tract infections. Indeed, fimbrial expression was found to be highly upregulated in the bacterial population in urine and infected bladders and downregulated in the lungs. In this organ, expression of type 1 fimbriae may be a disadvantage for the bacteria because of their ability to adhere to phagocytic cells in the lungs and therefore to be rapidly eliminated [106,107,108]. Moreover, FimH was shown to be required for Kp invasion and biofilm formation in a murine model of UTI [109] but the role of type 1 fimbriae in colonization of the gastrointestinal tract still remains controversial. Indeed, while Jung et al. observed the decreased colonization ability of a Kp *fimD* mutant of antibiotic-treated mice intestine (FimD is appointed to facilitate assembly and translocation of the pilus), another recent work showed data supporting that type 1 fimbriae do not contribute to gastrointestinal colonization as no significant difference in adhesion was noticed between wild-type and *fimH* mutant strains [110,111].

The components of type 3 fimbriae are encoded by the genes in the *mrkABCDF* operon [112,113]. The *mrk* gene cluster, as other fimbrial operons of the chaperone-usher class, contains genes encoding the chaperone (*mrkB*), the usher (*mrkC*) and the protein-based filament composed by a major (*mrkA*) and a minor (*mrkF*) subunit. MrkD is the tip adhesion protein, which is appointed for the adhesive properties of the whole complex, whereas to MrkE has been attributed a regulatory activity [112,114]. 

Among different *Klebsiella* strains, it is possible to find a plasmid-borne determinant, *mrkD*_1P_, and a chromosomally borne gene, *mrkD*_1C_, which are not genetically related, and the proteins encoded have been shown to have differences in functionality. MrkD adhesin located within a chromosomally borne gene cluster mediates binding to collagen types IV and V, whereas only few strains presenting the plasmid-borne form showed collagen-binding activity [115].

The presence of *mrk* genes in multiple genomic locations, including conjugative plasmids or transposons, can explain why this operon is so widespread among Gram-negative bacteria [116,117].

Type 3 fimbriae have been extensively proven to mediate binding to extracellular matrix (ECM) proteins such as collagen molecules [115,118] and to strongly promote biofilm formation [119,120,121]. 

A recent study revealed that MrkA expression was downregulated after treatment of Kp with the phytosynthesized silver nanoparticles (AgNPs) fabricated from *Mespilus germanica*, as demonstrated by RT-PCR analysis. In correlation with MrkA expression, also a strong reduction in biofilm formation was observed following treatment with AgNPs, confirming once again the leading role played by MrkA in this process [122]. Interestingly, investigation of MrkA regulation by Wilksch et al. has led to the understanding of another key player in type 3 fimbriae regulation, named MrkH, which directly activates transcription of the *mrkA* promoter. MrkH strongly binds to the *mrkA* regulatory region only in the presence of c-di-GMP, a second messenger molecule known to regulate the expression of many bacterial factors involved in colonization and biofilm formation [123,124,125,126,127].

### 3.4. Siderophores

Siderophores are secreted small molecules that can bind the iron present externally and re-enter the bacterial cells through specific receptors [128,129]. Kp production of siderophores such as enterobactin, yersiniabactin, salmochelin, and aerobactin has been shown to strongly correlate with in vivo virulence and differentiate Hv-Kp from classical Kp strains. For this reason, siderophores have been suggested as potential biomarkers to be further implemented in screening laboratory tests [29,130,131,132]. Each of these molecules presents a specific receptor on the bacterial surface able to bind the siderophore and to mediate the iron uptake, which is essential for the pathogen survival. 

## 4. Vaccines and Monoclonal Antibodies (mAb) Strategies

No vaccines are currently licensed against Kp, but several vaccine targets have been described in the past few decades [133]. Moreover, a therapeutic approach based on the use of monoclonal antibodies (mAbs) against AMR pathogens has been taking place over the last years [134].

Several research models have been implemented in the processes of vaccine development or mAbs identification, chiefly rats and mice. Vaccine antigens or mAbs have been tested in the mouse model to investigate two clinical manifestations of *Klebsiella* infections: pneumonia and sepsis. The mouse pneumonia model recapitulates key features of *Klebsiella*-induced pneumonia in humans, characterized by a massive inflammation with influx of polymorphonuclear neutrophils and oedema. However, the results obtained with this model have uncovered many aspects implicated in host defense against the pathogen, due to the differences between mice and humans in immune system development and response to infection [135,136]. More recently, other models are proving to be useful in assessing Kp infection biology, such as the non-mammalian models *Galleria mellonella* and *Danio rerio* (zebrafish) that are more easy to handle and still recapitulates interaction with the innate immune system [136]. Certainly, non-human primates, which are also naturally infected by Kp, would provide the best model for the investigation of vaccines/mAbs efficacy, especially squirrel monkeys that were described to better mimic human respiratory pneumonia compared to rodents, but so far have been only employed to test antibiotics [137,138].

### 4.1. K-Antigen Based Approaches

Bacterial capsule polysaccharides have been used historically as vaccine target antigens and several formulations have been developed against different pathogens and licensed worldwide in the last 40 years [139]. However, a K-antigen-based immunotherapeutic strategy against Kp is complicated because of the high variability and structural diversity. 

Different preparations of K-antigen vaccines have been tested in preclinical and clinical studies and used to produce hyperimmune human sera as a therapeutic. One of the first tries dates back to 1982, when serum recovered from mice immunized with UV-irradiated Kp was added to the inoculum of bacteria used to infect the animals, producing 50% of mice protection. In the study, a relationship between protective ability and hemagglutinating activity of the serum was demonstrated, which indicates the presence of antibodies directed against the capsule [140]. 

Several published studies have shown the ability of anti-K antibodies to confer protection against Kp in animal models of infection. Cryz et al. demonstrated that rabbit anti-K antibodies were able to reduce mortality when administered to mice before challenge with homologous Kp [141]. The same authors later showed that anti-K1 IgG isolated from human volunteers are able to protect mice from Kp sepsis [142]. Additionally, a murine anti-K2 IgM monoclonal antibody was tested in rats against experimental Kp challenge and, even though the invasion was not prevented, mAb-treated animals showed a more rapid bacterial clearance resulting in an accelerated resolution of infection [143]. In 1985, human volunteers were vaccinated using a preparation of K1 polysaccharide detoxified from trace quantities of LPS by treatment with 95% ethanol-0.1 N NaOH solution. This treatment reduced local reactions compared to the control group vaccinated with the untreated K1 and all vaccinees responded with a fourfold or greater rise in IgG and IgM titers. These antibodies were further proven to prevent Kp sepsis when tested in mice [142]. 

Considering that the ideal capsule-based vaccine should be multivalent in order to cover the majority of all bacteraemic isolates, Cryz et al. subsequently tested a polyvalent *Klebsiella* vaccine, composed of six K-serotypes (K2, K3, K10, K21, K30, and K55), which resulted to be safe and immunogenic in humans [144,145]. However, a following epidemiological study raised the awareness that a Kp vaccine able to cover around 70% of clinically relevant Kp strains should include at least 25 capsular polysaccharides [146]. With this aim, the same group formulated a vaccine consisting of 24 unconjugated K-antigens, manufactured by the Swiss Serum and Vaccine Institute, together with a *Pseudomonas aeruginosa* vaccine consisting of eight O-antigen polysaccharides conjugated to *Pseudomonas* toxin A. This formulation showed safety and immunogenicity in a phase 1 human trial and allowed one to collect hyperimmune globulin for intravenous use (H-IVIG) from the plasma of vaccinees [147,148]. Although the administration of H-IVIG proved to be effective in reducing the severity of Kp infection, these results were not statistically significant, and many patients suffered from adverse reactions [149]. 

Other groups followed the conjugation approach to increase K-antigens immunogenicity. Already in 1985, an octasaccharide obtained from K11 was conjugated to either bovine serum albumin or to keyhole limpet hemocyanin and tested in wild type and nude mice. This work showed that the coupling of this oligosaccharide to proteins converted the antigen from T-independent to T-dependent [150]. More recently, another group identified a shared hexasaccharide repeating unit (hexasaccharide 1) in the K-antigens of CR-Kp isolates responsible for a deadly outbreak in 2011 and conjugated it to CRM_197_, obtaining a vaccine that resulted immunogenic in mice and rabbits [151]. 

An interesting study was performed in 2019 by Feldman et al. who tested for the first time a bioconjugate vaccine encompassing capsules from the K1 and K2 serotypes, causing around 70% of all Hv-Kp infections worldwide [152]. The bioconjugate vaccine, produced in a glycoengineered *E. coli* strain, was made by the transfer of K1/K2 polysaccharides from a lipid-liked precursor to a genetically deactivated exotoxin A from *Pseudomonas aeruginosa*. K1 and K2 bioconjugates elicited serotype-specific IgG responses in mice and were also able to protect animals from Hv-Kp infection, confirming this strategy to be very promising for the development of a Kp vaccine. For their relevance in protecting most of the Hv-Kp strains, K1 and K2 were also investigated as potential targets for mAbs. 

Recently, Diago-Navarro et al. isolated two mAbs, 17H12 and 8F12, targeting the above-mentioned hexasaccharide 1 and able to agglutinate several CR-Kp strains, presenting both K1, K2 and other K-serotypes. The functionality of these mAbs was extensively proven in vitro (e.g., revealing the ability to promote opsonophagocytic killing and neutrophil extracellular trap (NET) release), and in in vivo studies, where protection against K1 strains in three distinct murine infection models was demonstrated. The authors claimed that these two mAbs can be considered promising candidates for an antibody-based approach to the treatment of CR-Kp infections and that the hexasaccharide 1 should be further explored for vaccine development [153].

### 4.2. O-Antigen Based Approaches

Vaccination strategies against Kp have largely focused on the capsule polysaccharides. However, Kp O-antigens are alternative targets for the development of a vaccine, due to their limited range of structures. Indeed, four serotypes (O1, O2, O3 and O5) were predicted to cover over 80% of clinically relevant Kp strains [54,76,154,155]. Nevertheless, the development of O-antigen-based vaccines against Kp has not yet progressed beyond the preclinical phase. 

Clements et al. showed that immunization with purified O1 LPS vaccine could protect mice against K2:O1 challenges [155]. Additionally, Chhibber et al., reported that O1 LPS, incorporated either into liposomes or sodium alginate microparticles, could protect rodents against lobar pneumonia and resulted to be less toxic than free LPS in mice [156]. A year later, the same authors prepared a glycoconjugate vaccine using the O1 O-antigen covalently linked to tetanus toxoid: the conjugate was found to be non-pyrogenic in rabbits and immunoprotective, as verified from the decrease in the relative colonization of bacteria in lungs of immunized rats as compared to the control animals [157]. 

Recently, Hegerle et al. reported the development of a combined Kp and *Pseudomonas aeruginosa* glycoconjugate vaccine comprising O1, O2, O3, O5 Kp O-serotypes, chemically linked to the two *Pseudomonas* flagellin types (FlaA, FlaB) [158]. The quadrivalent conjugate vaccine generated antibody titers to the four Kp O-antigens and both Fla antigens in rabbits. Innovative strategies have been recently proposed for the generation of O2a glycoconjugate vaccines including a semi-synthetic approach and a bioconjugation approach [159,160]. 

Of note, anti-Kp O-antigen mAbs also proved to be able to reduce bacterial burden, enhance survival, and show synergy with current standard of care therapy [161]. Pennini et al. demonstrated that the serotype specific anti-O1 (KPE33) and anti-O2 (KPN42) human mAbs are able to protect mice against Kp infection via opsonophagocytic killing [58]. Guachalla et al., generated an anti-O3 mAb 2F8, able to cross-react with all three O3 sub-serogroups (O3, O3a and O3b) [162]. 

### 4.3. Protein Based Approaches

Protein virulence factors have also been proposed as targets for the development of vaccines and therapeutic mAb against Kp. These antigens are characterized by low variability, especially when compared to the K-antigens and O-antigens polysaccharides.

In particular, type 3 fimbriae structural protein MrkA has gained a strong interest as a putative target for a vaccine as, besides being expressed by most of Kp strains, including Hv-Kp, it shows a structural position on the pilus filament that may ensure easy access to antibodies. There are already data showing that immunization with purified fimbriae was able to protect mice against a lethal challenge in a model of acute pneumonia [163]. In 2016, Wang et al., immunized mice subcutaneously with both monomeric and oligomeric MrkA formulated with Freund’s adjuvant and the vaccinated mice showed a reduction in the organs’ bacterial burden after intranasal challenge with Kp [164]. In the same work, using high throughput opsonophagocytic killing (OPK) screens, the authors identified an anti-MrkA mAb, named KP3, able not only to determine OPK activity against different Kp serotypes, but also to reduce Kp adhesion to human pulmonary epithelial cells A549. Interestingly, these data also showed that OPK may be a reliable assay to measure in vitro antibodies’ ability to mediate in vivo protection [164]. 

Type 1 and 3 fimbriae were used as carrier proteins conjugated to *E. coli* core-oligosaccharide, proving to be immunogenic also in rabbits [165]. Furthermore, Cross and coauthors have recently submitted a patent on a multiple antigen presenting system (MAPS) vaccine encompassing MrkA conjugated to four Kp O-antigens (O1, O2, O3 and O5), providing experimental evidence of good immunogenicity in mice [133].

Recently, using bioinformatic prediction tools, Zargaran et al., found four B cell epitopes (each for one Fim antigen) that were proposed as suitable vaccine candidates for Kp as these epitopes may be recognized by the B cell receptor, triggering humoral responses. Indeed, in silico analysis suggested that these epitopes are immunogenic and antigenic, not similar to human peptides, not allergenic and not toxic. However, these results still need to be supported by in vitro and in vivo testing [166].

Dar et al., in 2019, applied a reverse vaccinology approach on a total of 222 available complete genomes of Kp and four outer membrane proteins were shortlisted for vaccine designing: the outer membrane protein OmpA, the copper/silver efflux RND transporter, the phosphoporin PhoE and the peptidoglycan-associated lipoprotein Pal. Of these antigens, a total of four epitopes were joined together using flexible linkers resulting in a potential multi-epitope vaccine against Kp [167].

Another virulence factor recently investigated as a potential vaccine target is the highly conserved YidR, an ATP/GTP-binding protein that mediates the hyperadherence phenotype and is involved in biofilm formation [168]. Recombinant YidR has been proven to protect mice from challenge with a low dose of Kp [168].

## 5. Conclusions

The use of antimicrobials has historically enabled the treatment of life-threatening diseases, however the global increase in AMR has compromised the ability to cure a wide range of infections and has been paralleled by a general slowdown in the development and commercialization of novel antibiotics [169]. 

*Klebsiella pneumoniae* is a genetically and phenotypically variable bacterium which has become a significant threat to public health over the past few decades. MDR-Kp lineages, such as CG258, have evolved resistances to carbapenems and are responsible for hospital-acquired infections worldwide. Hv-Kp lineages, such as CG23, are instead associated with community-acquired invasive infections.

In both cases, novel strategies for prophylaxis and treatment of infections are urgently needed, including vaccines and monoclonal antibodies. Indeed, the use of mAbs could be advantageous to protect against hospital-acquired infections in patients at high risk, compared to vaccination. A mAb-based approach would be potentially attractive particularly for immunocompromised or elderly subjects, who may respond sub-optimally to vaccination. On the other hand, a vaccination approach would be more suitable to prevent community-acquired or hospital-acquired infections, especially in LMIC, where maternal immunization could potentially protect newborns from hospital acquired infections leading to neonatal sepsis. 

There are, however, some barriers in developing vaccines and mAbs against Kp infections. First of all, the great serotype variability requires either high-valency vaccines or mAbs cocktails to obtain acceptable coverage. K-antigens have been historically considered key protective antigens for both active and passive immunization. However, less diverse polysaccharides as Kp O-antigens or highly conserved surface proteins as fimbriae are recently gaining attention as alternative approaches. While there are growing evidences supporting the ability of anti-Kp O-antigens and anti-fimbriae mAbs to protect mice in therapeutic models, there are still concerns around the protective immunity conferred by antibodies to sub-capsular antigens, especially considering the hypercapsule production in Hv-Kp. A second limitation is represented by the available in vivo models. Indeed, differences have been highlighted in Kp pathogenicity between humans and animal models (e.g., the CR-Kp CG258 is responsible for serious infections in humans but is rapidly cleared in mice and rats [170]). Finally, in vitro models need to reflect the diverse mechanisms operating in vivo to prevent infection and should allow the understanding of how mAbs work in the context of the immune system. 

## 6. Future Perspectives

Preventive vaccines as well as therapeutic mAbs represent complementary approaches to fight AMR. Innovative technologies are needed to tackle complex pathogens such as *Klebsiella*, by targeting multiple antigens at the same time. 

Recently, outer membrane vesicles (OMVs) from Kp were proposed as a vaccine candidate and conferred protection in a preclinical animal model, with a mechanism dependent on both humoral and cellular immunity [171]. This result emphasizes the need of novel strategies combining polysaccharide and protein antigens in a single formulation [172]. Additionally, optimal delivery systems need to be identified according to the target population, including a preference for single dose vaccination for use in pregnant women.

In parallel, improvements in the field of mAbs (e.g., new technologies enabling the generation of bispecific mAb) could result in passive administration of few unique mAbs (e.g., 2–3 mAbs) targeting key virulence factors and providing protection against the vast majority of clinical isolates [173].
